# The ENG/VEGFα Pathway Is Likely Affected by a Nonsense Variant of Endoglin (ENG)/CD105, Causing Hereditary Hemorrhagic Telangiectasia Type 1 (HHT1) in a Chinese Family

**DOI:** 10.3390/genes15030304

**Published:** 2024-02-27

**Authors:** Kemeng Liu, Jiewen Fu, Kan Guo, Mazaher Maghsoudloo, Jingliang Cheng, Junjiang Fu

**Affiliations:** 1 Key Laboratory of Epigenetics and Oncology, The Research Center for Preclinical Medicine, Southwest Medical University, Luzhou 646000, China; liukemeng@swmu.edu.cn (K.L.); fujiewen@swmu.edu.cn (J.F.); guokan@swmu.edu.cn (K.G.); mazaher@swmu.edu.cn (M.M.); jingliangc@swmu.edu.cn (J.C.); 2 School of Basic Medical Sciences, Southwest Medical University, Luzhou 646000, China

**Keywords:** hereditary hemorrhagic telangiectasia type 1 (HHT1), endoglin (ENG), whole exome-sequencing (WES), nonsense mutation, bleomycin, VEGFα, TGFβ

## Abstract

Hereditary hemorrhagic telangiectasia (HHT), also called Rendu–Osler syndrome, is a group of rare genetic diseases characterized by autosomal dominance, multisystemic vascular dysplasia, and age-related penetrance. This includes arteriovenous malformations (AVMs) in the skin, brain, lung, liver, and mucous membranes. The correlations between the phenotype and genotype for HHT are not clear. An HHT Chinese pedigree was recruited. Whole exome sequencing (WES) analysis, Sanger verification, and co-segregation were conducted. Western blotting was performed for monitoring ENG/VEGFα signaling. As a result, a nonsense, heterozygous variant for ENG/CD105: c.G1169A:p. Trp390Ter of the proband with hereditary hemorrhagic telangiectasia type 1 (HHT1) was identified, which co-segregated with the disease in the M666 pedigree. Western blotting found that, compared with the normal levels associated with non-carrier family members, the ENG protein levels in the proband showed approximately a one-half decrease (47.4% decrease), while levels of the VEGFα protein, in the proband, showed approximately a one-quarter decrease (25.6% decrease), implying that ENG haploinsufficiency, displayed in the carrier of this variant, may affect VEGFα expression downregulation. Pearson and Spearman correlation analyses further supported TGFβ/ENG/VEGFα signaling, implying ENG regulation in the blood vessels. Thus, next-generation sequencing including WES should provide an accurate strategy for gene diagnosis, therapy, genetic counseling, and clinical management for rare genetic diseases including that in HHT1 patients.

## 1. Introduction

Hereditary hemorrhagic telangiectasia (HHT), also called Rendu–Osler syndrome, is a rare genetic disease characterized by autosomal dominance, multisystemic vascular dysplasia, and age-related penetrance. This includes arteriovenous malformations (AVMs) in the skin, brain, lung, liver, and mucous membranes [1,2]. The current prevalence estimates for HHT are approximately 1 in 5000 worldwide [3]. AVMs can occur anywhere in the body but they most appear on the lips, face, tongue, nasal area, fingertips, buccal region, as well as gastrointestinal mucosa. Because of their thin walls, vessels that are close to the skin or mucosal surfaces are prone to rupture and bleeding from minimal injury. Hallmark features are epistaxis and mucocutaneous telangiectasias. Recurrent epistaxis frequently occurs due to telangiectases of the nasal mucosa, especially in older HHT patients.

Three genes have been identified as disease-causative genes responsible for HHT disorder. These are endoglin (ENG, or CD105; OMIM: 131195) on chromosome 9q34.11 [4], activin receptor-like kinase 1 (ALK1; OMIM: 601284) on chromosome 12q13.13 [5], and SMAD family member 4 (SMAD4; OMIM: 600993) on chromosome 18q21.2 [6]. Clinically, hereditary hemorrhagic telangiectasia type 1 (HHT1; OMIM: 187300) is caused by mutations of the *ENG* gene [4], whereas hereditary hemorrhagic telangiectasia type 2 (HHT2; OMIM: 600376) is caused by mutations of the *ALK1* gene. Pathogenic mutations in the genes for *ENG* and *ALK1* account for approximately 90% of HHT patients, specifically causing HHT1 and HHT2 diseases, respectively. The other three genes, GDF2 (OMIM: 605120) [7,8], RASA1, and EPHB-4, have been reported to be associated with overlap diseases that create an HHT-like phenotype [2].

The *ENG* gene, also called CD105, encodes a glycoprotein as a homodimeric transmembrane in the vascular endothelium. ENG associates with the human vascular endothelium and also presents on bone marrow proerythroblasts for activating the monocytes and lymphoblasts of childhood leukemia. This protein complexes with the transforming growth factor β (TGFβ) receptor (the β1 and β3 peptides) for high affinity [9]. The VEGF level correlates with TGF-β levels. Recent reports have shown that NRP1, a cellular surface receptor for SARS-CoV-2 [10], can interact with ENG and VEGFR2 to regulate VEGF signaling and endothelial cell sprouting [11]. In addition, ENG may be affected by the BMP9-mediated pathway [12,13]. This gene may also play roles in pre-eclampsia and cancers.

ENG is mainly expressed in endothelial cells and endothelial colony-forming cells, serving as a biomarker of angiogenesis [14]. Alternatively, spliced transcript variants (transcript variants 1 and 2) of *ENG,* encoding various isoforms, have been reported, and both variants play roles in activating monocytes, endothelial cells, and placenta, with the longer isoform being predominant [15]. Isoform 1 encodes 658 amino acids with a predicted molecular weight of 70,578 Da, while isoform 2 encodes 625 amino acids with a predicted molecular weight of 67,542 Da. The differences between isoforms 1 and 2 lie in the C-terminus of ENG, where 618 amino acids are identical to the N-terminus of ENG (isoform 1: NP_001108225.1, isoform 2: NP_000109.1).

The diagnosis for HHT is clinically mainly derived from the so-called Curaçao (Shovlin) criteria [16]. Given the largely variable onset of disease phenotype and the age-related penetrance, however, these Curaçao criteria show low sensitivity in at-risk children and adolescents during the first decade of life [2,17]. If lacking a reliable clinical history, the diagnosis of HHT becomes challenging, especially in pediatric patients, based on the Curaçao criterion. In addition, patients who received symptomatic treatment at primary-care centers might lack a clinical awareness of HHT, and few will be referred to specialized HHT centers for further management [18]. The multidisciplinary coordinated care and management of HHT are deemed necessary [19]. Since visceral AVM can have serious consequences if left undiagnosed and untreated, a delayed diagnosis might lead to a failure in establishing timely intervention/management, resulting in increased clinical harm, particularly in pediatric patients, due to the reduced sensitivity of the clinical criteria in this age group [20,21]. Moreover, the correlations between phenotype and genotype for HHT are not clear. With great variabilities in phenotypes among HHT patients, genetic diagnosis may play a critical role when triaging patients, particularly at an early stage. Next-generation sequencing (NGS), such as whole exome sequencing (WES) and target sequencing [22], should offer an important strategy for the clinical diagnoses of HHT patients.

## 2. Materials and Methods

### 2.1. Patient Information and Sample Collection

**Figure 1:**
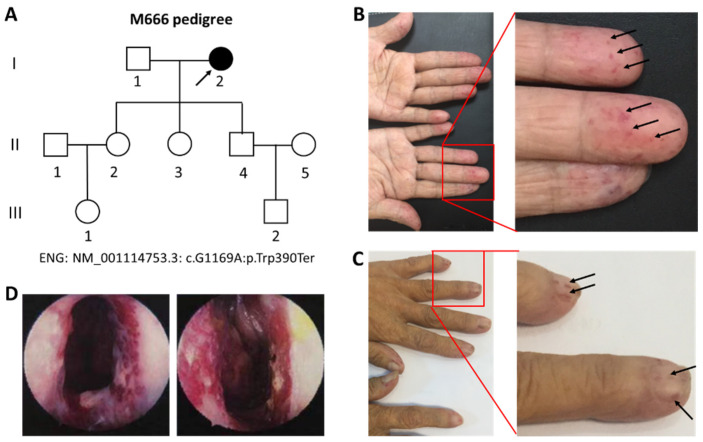
An M666 pedigree with hereditary hemorrhagic telangiectasia type 1 (HHT1). (**A**). M666 pedigree with hereditary hemorrhagic telangiectasia type 1 (HHT1). Unaffected individuals are shown as clear circles for females or squares for males. The filled circle indicates the proband (I:2, arrow) with the ENG mutation-ENG: NM_001114753.3; exon9; c.G1169A:p.W390X. (**B**). The phenotype of hereditary hemorrhagic telangiectasia in the fingers of the proband (I:2). (**C**). The phenotype of hereditary hemorrhagic telangiectasia in the nails of the proband (I:2). Right panels of (**B**) and are the enlarged images of the left panels. (**D**). The images are derived from the ENT laryngoscope.

The patient/proband (Figure 1A, I:2, M666) was a 66-year-old female who had experienced repeated nasal and lip bleeding for more than 40 years, with an exacerbation over the last 10 years. Blood coagulation and chemistry examinations were also performed. Written informed consent was provided to enroll, and the study was approved by the Ethics Committee of Southwest Medical University.

Blood samples were collected, including from the proband and her 8 family members. DNA was extracted using proteinase K digestion and phenol/chloroform treatment [23,24]. Simultaneously, the whole blood proteins were lysed from the proband and her 5 family members for Western blotting.

### 2.2. WES Analysis

Whole-exome sequencing (WES) analysis of the gDNA of the proband M666 was performed as described previously [25,26,27]. After library preparation, hybridization, PCR amplification, purification, and cluster generation, an NGS assay was performed on the Illumina instrument (Illumina, Inc., San Diego, CA, USA), utilizing dual-end sequencing mode. The sequencing covered a 47 Mb genomic exon sequence, achieving an average data detection depth of more than 100, which efficiently detected SNP and Indel mutations. The sequencing result reads were aligned to the known human reference genome sequence GRCh37/hg19 (UCSC) using BWA (Burrows-Wheeler Aligner) software package, and the variant sites were annotated by ANNOVAR using the GATK 3.1.1 variant-detection software. Interpretation reports prioritized mutations that are currently clearly or possibly associated with the diseases. Unless relevant pathogenicity had been reported, the outcome of screening excluded polymorphic mutations such as synonymous mutations, non-decoding frame mutations, sites with low coverage, intronic regions, and non-coding RNA regions. All data were interpreted based on the current knowledge of diseases and causative genes. The mutation sites were annotated and compiled in a file [26,28].

### 2.3. Sanger Verification and Co-Segregation Analysis

The ENG primers for Sanger verification and co-segregation analysis were designed as follows: ENG-1587L-ctgggttgtggtcagtcctt; ENG-1587R-ctgctctcccaaacacacct. A 10 μL volume was used for PCR amplification. The PCR product size was 326 bp. After successful PCR amplification, the product was subjected to Sanger sequencing using the primer ENG-1587L on an ABI 3500DX Genetic Analyzer (ABI, USA) [29].

### 2.4. Expression Analysis for ENG in Different Tissues and Different Types of Blood Cells in H. sapiens

ENG expressions were analyzed in Human Protein Atlas (HPA) data from different human tissues (https://www.proteinatlas.org/ENSG00000106991-ENG/tissue) (accessed on 1 January 2024) and different types of human blood cells (https://www.proteinatlas.org/ENSG00000106991-ENG/blood) (accessed on 1 January 2024) [30,31,32,33].

### 2.5. Western Blotting

The endoglin/CD105 polyclonal antibody was purchased from Wuhan Sanying (rabbit polyclonal, catalog number: 10862-1-AP, Proteintech Group, Inc., Rosemont, IL, USA). The mouse monoclonal anti-VEGFα (vascular endothelial growth factor α) antibody was purchased from abcam (VG-1, Cat # ab1316, abcam, Cambridge, UK). Western blotting was performed for the proband and her 5 family members. β-actin was used as an internal control. The endoglin/CD105 antibody and VEGFα antibody were used at 1:4000 dilutions, while β-actin was used at a 1:5000 dilution.

### 2.6. Correlation Analysis

The Genotype Tissue Expression (GTEx) expression data were derived from the GTEx project, a comprehensive public resource that studies tissue-specific gene expression and regulation. The data were primarily generated for genetic analysis, including via WGS, WES, and RNA-Seq, using human samples which were collected from 54 non-diseased tissue sites in approximately 1000 individuals [34]. We utilized whole blood and artery samples (aorta, coronary, and tibial). Initially, we transformed the datasets into log2(TPM) and then focused on the expression of ENG, VEGFA, and TGFβ. Subsequently, we computed the correlation between ENG and VEGFA, as well as ENG and TGFB, in both blood and artery samples. Spearman and Pearson correlation coefficients were employed to illustrate linear regression and monotonic curve plots. The plots were generated using the ggplot2 (ver. 3.4.4) R-4.3.1 package.

## 3. Results

### 3.1. Patient Clinical Information and Pedigree Recruitment

As of 40 years ago, there was no obvious cause for the proband-repeated bilateral nasal and lip bleeding in the proband (Figure 1A, I:2, M666), which can be relieved by packing and compression; 10 years ago, the above symptoms were aggravated, and the nasal cavity and lips were repeatedly bleeding, so it was necessary to fill their nose in the hospital and apply local electrocoagulation to the nasal cavity, along with hemostasis, but the treatment was not effective. She complained of bleeding at different degrees every week, and anemia was treated via blood transfusion. An ENT laryngoscopy was performed. Clinical examinations of the proband (Figure 1A, I:2) showed that the skin and mucosa displayed bright red or purple-red blood vessels with a diameter of 1~3 mm, which is a needle tip-sized mass, or hemangiomas. Figure 1B shows the red blood vessels along with the mass diameter phenotype of the fingers of the proband (Figure 1B, arrows), while Figure 1C shows the needle tip-sized red blood vessels in the nails of the proband (Figure 1C, arrows). The ENT examination indicated bilateral erosion of the nasal mucosa; white secretions were noted bilaterally, and the blood vessels showed a mass of proliferation, but the bilateral inferior turbinate showed no hypertrophy (Figure 1D, M666). Blood chemistry examinations in the proband showed that the levels of total bilirubin (TBIL), direct bilirubin (DBIL), and indirect bilirubin (IBIL) were abnormally upregulated (Table 1), while blood coagulation examination and platelet counts showed normal results (Table 2). Other family members displayed no relevant HHT-related clinical issues (Figure 1). The mother of the proband died when she was a child and her father died when she was a teenager, so she was not aware of similar symptoms. We thus primarily clinically diagnosed hereditary hemorrhagic telangiectasia (HHT) in the proband as she met three of the Curaçao criteria (Table 3) [16]. Blood samples were collected from the proband (Figure 1) and the eight family members, and DNA was also extracted for further genetic analysis.

### 3.2. Identifying a Heterozygous Variant for ENG: c.G1169A:p. Trp390Ter of the Proband with HHT1

**Figure 2:**
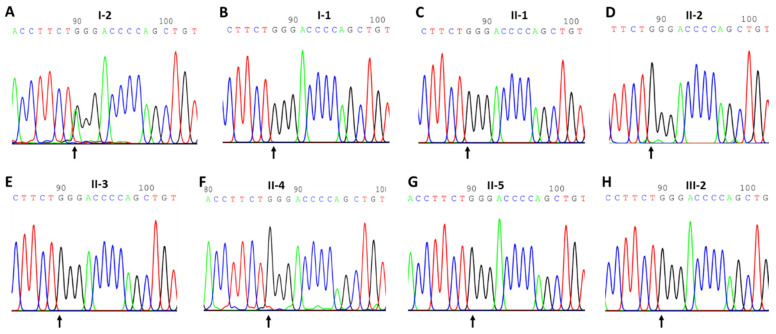
Sanger sequencing profiles in the M666 family. (**A**). The sequenced results in I-2 (heterozygous mutant type) for ENG: NM_001114753.3: exon9: c.G1169A:p.W390X. (**B**). The sequenced results are in I-1 (wild-type). (**C**). The sequenced results are in I-2 (wild-type). (**D**). The sequenced results are in II-2 (wild-type). (**E**). The sequenced results are in II-3 (wild-type). (**F**). The sequenced results in II-4 (wild-type). (**G**). The sequenced results are in II-5 (wild-type). (**H**). The sequenced results are in III-2 (wild-type). The arrows show the mutant position.

The whole-exome sequencing (WES) of the proband showed that the target area coverage was 99.48%, the average depth of the target region was 105.25, and the target region’s mean depth > 20 ratio was 98.70%. After analysis, we identified a nonsense, heterozygous variant c.G1169A with a nucleotide change from G to A in exon 9 of the *ENG* gene (NM_001114753.3), leading to the production of a stop codon “TAG” from “TGG”, and amino acid exchanges Trp (W) at position 390 to a stop codon Ter (X) (p.Trp390Ter) in the ENG protein (NP_001108225.1) (Figure 1A, II:1). The ENG c.G1169A mutation was verified by Sanger sequencing (Figure 2A). This mutation was absent in the 100 ethnically matched normal controls. Thus, our studies suggest that the *ENG* pathogenic mutation should cause the HHT1 disease with pathogenicity in this patient from a Chinese family (American College of Medical Genetics and Genomics (ACMG) evidence: PVS1 + PM2 + PP5), according to the criteria of ACMG and the Association for Molecular Pathology guidelines (AMP) [35,36]. The mutation did not include in the 1000 Human Genome Project, gnomAD, and ExAC, or the Chinese millionome database, but it was seen in the ClinVar database [37]; thus, it may be novel in Chinese patients. Based on the genetic diagnostic results, which yielded a variant which is predicted to encode an ENG transcript bearing a premature stop codon, this patient was ascertained as having hereditary hemorrhagic telangiectasia type 1 (HHT1).

Due to the premature termination codon, the mutant transcript is likely to be degraded through the nonsense-mediated decay (NMD) mechanism, thus leading to a lack in the predicted mutant ENG polypeptide synthesis [38]. As a consequence, haploinsufficiency and loss-of-function of ENGs are likely to result in impaired ENG signaling and HHT-associated pathway alteration [4,14].

### 3.3. Segregation for ENG: c.G1169A in the M666 Pedigree

For the segregation analysis, we then conducted Sanger sequencing on the members of the M666 family. The proband’s ENG nonsense mutation was absent in all analyzed relatives, i.e., the proband’s husband, three children, the proband’s children’s spouses, and the proband’s grandson (Figure 2B–H). Family member III:1 is a university student who refused genetic testing. Thus, segregation analysis was conducted and demonstrated that all the M666 members we tested in this pedigree are normal, which is consistent with the clinical observations for these family members.

### 3.4. Wild-Type ENG Expression in Tissues and EMG Mutant Form in the Proband

**Figure 3:**
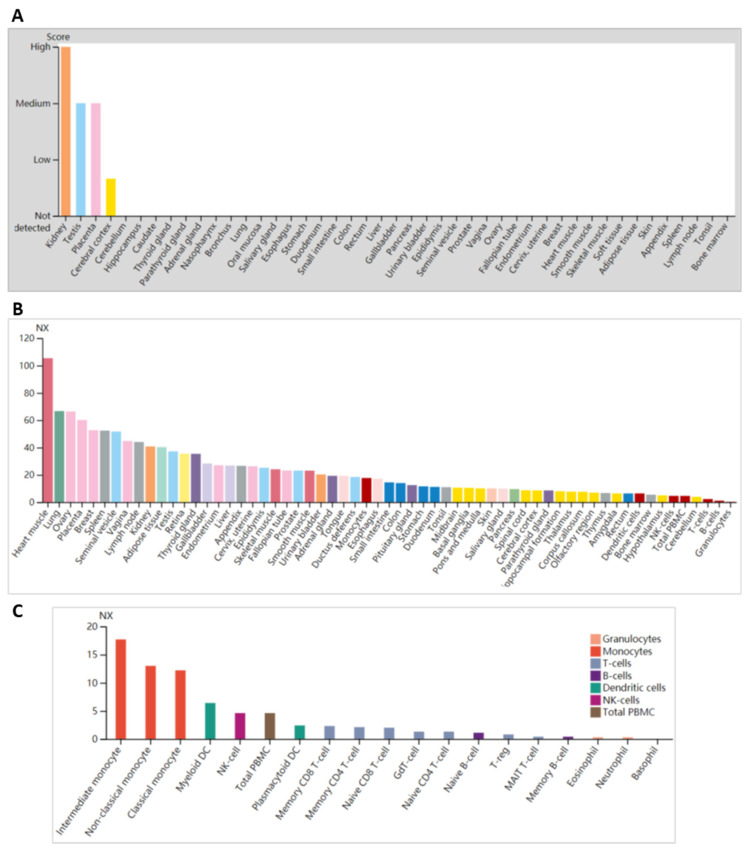
ENG expression in tissues or cells from humans. (**A**). ENG protein expression in different human tissues (indicated). (**B**). *ENG* mRNA expressions in different human tissues (indicated) (**C**). *ENG* mRNA expressions in different indicated blood cells. NX, normalized expression.

The analysis for ENG protein expression based on HPA data in different human tissues found that ENG is most highly expressed in the kidneys, then in the testis and placenta, but is low in the cerebral cortex; no expression was detected in the other 40 tissues (Figure 3A). Analyses for *ENG* mRNA expressions based on RNA data in different human tissues showed that *ENG* has low tissue specificity and is most highly expressed in the heart muscle (105.4 NX), then in the lung (66.7 NX), ovary (66.4), and placenta (60.2 NX). Its presence in granulocytes was the lowest, at 0.3 NX in 55 tissues (Figure 3B). In order to examine ENG’s expression level, we analyzed blood samples and found that relatively low levels, mainly in the intermediate monocyte (17.7 NX), non-classical monocyte (13 NX), classical monocyte (12.2 NX), myeloid DC (6.4 NX), NK-cell (4.6 NX), and total peripheral blood mononuclear cells (PBMC) (4.6 NX), derived from 18 blood-cell types and one PBMC (Figure 3C), which may be known in the HHT scientific community for the low-level expression of ENG in most circulating cells. Nevertheless, we performed Western blotting on the blood samples taken from the proband and her family.

### 3.5. ENG Variant May Affect TGFβ/VEGFα Signaling

**Figure 4:**
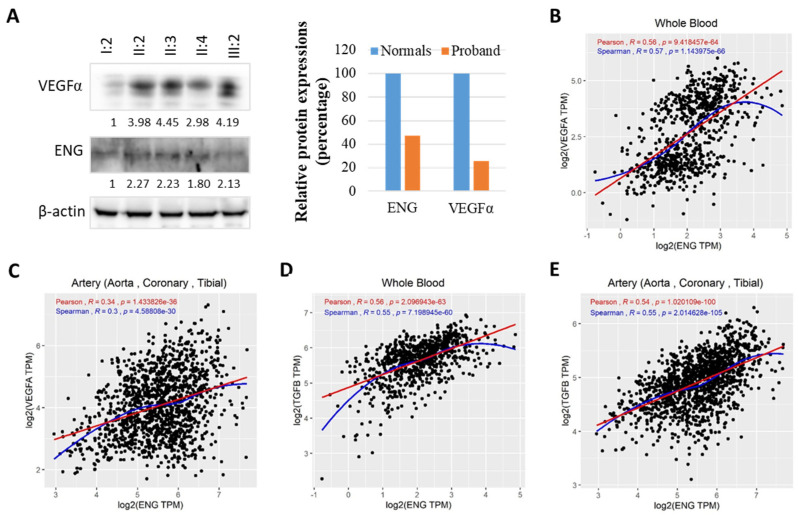
The ENG variant may affect TGFβ/VEGFα signaling. (**A**) Western blotting results depicts the expression of ENG and VEGFα in the proband and family members using blood samples. Left panel shows Western blotting results, while right panel is quantitative results for Western blotting. (**B**) Pearson and Spearman correlation analyses between the ENG and VEGFα genes’ GTEx expression data in the blood of humans. (**C**) Pearson and Spearman correlation analyses between the ENG and VEGFα genes’ GTEx expression data in arteries (aorta, coronary, and tibial). (**D**) Pearson and Spearman correlation analysis of the genes between ENG and TGFβ (TGFB) in blood of GTEx expression data, respectively. (**E**) Pearson and Spearman correlation analysis of the genes between ENG and TGFβ (TGFB) in arteries (aorta, coronary, and tibial) of GTEx expression data, respectively. *p* < 0.05 was considered to be significant.

The TGF-β signaling cascade, or TGF-β/ALK1/endoglin (ENG) signaling, promotes the proliferation, migration, and tube formation of endothelial cells. The VEGF level has also been reported to correlate with TGF-β levels. During the angiogenic process, a decrease in the ENG levels in HHT endothelial cells may interfere with the building of cord-like structures [39]. Then, we monitored the ENG and downstream VEGFα gene for angiogenesis via Western blotting using whole-blood lysates, and the results are shown in Figure 4A. Compared with the proband M666 patient (I:2), the ENG protein levels in family members II:2, II:3, II:4, and III:2 showed approximately two-fold increases (Figure 4A, left), while the levels of the VEGFα protein, a protein that promotes neovascularization and increases vascular permeability, showed approximately 3- to 4.5-fold increases in family members II:2, II:3, II:4, and III:2 (Figure 4A, left). In other words, when compared with the normal family members II:2, II:3, II:4, and III:2, the ENG protein levels in the proband M666 patient (I:2) were approximately halved (47.4% lower) (Figure 4A, right), while levels of the VEGFα protein were approximately one-quarter lower (25.6% decrease) (Figure 4A, right), demonstrating that the ENG protein haploinsufficiency caused by the ENG variant (p.Trp390Ter) in the proband may downregulate TGFβ/VEGFα expression in peripheral blood mononuclear cells (PBMC), implying ENG/TGFβ/VEGFα signaling. Western blot investigations clearly showed that the proband displayed a one-half reduction of the wild-type ENG protein compared to her family members, as expected due to NMD-mediated mutant transcript degradation. Accordingly, we did not observe the mutant polypeptide form of the ENG variant (p.Trp390Ter) in the proband, as a consequence of NMD degradation.

Moreover, Pearson and Spearman correlation analyses between ENG and VEGFα and between ENG and VEGFα in both blood and arteries were performed. We first analyzed the blood, and found a significantly positive correlation between ENG and VEGFα (Figure 4B, R = 0.56, *p*-value = 9.42 × 10^−64^ and R = 0.57, *p*-value = 1.14 × 10^−66^, respectively), supporting the Western blotting results in Figure 4A. We then analyzed the results in arteries, including the aorta, coronary, and tibial (blood vessels), and also found a significantly positive correlation between ENG and VEGFα (Figure 4C, R = 0.34, *p*-value = 1.4 × 10^−36^ and R = 0.3, *p*-value = 4.59 × 10^−30^, respectively), implying ENG/VEGFα regulation in the blood vessels. Similar results further showed significantly positive correlations between ENG (Figure 4D, R = 0.56, *p*-value = 2.096943 × 10^−63^ and R = 0.55, *p*-value = 7.198945 × 10^−60^, respectively) and TGFβ, and between ENG and TGFβ, in the blood or artery (Figure 4E, R = 0.54, *p*-value = 1.020109 × 10^−100^ and R = 0.55, *p*-value = 2.014628 × 10^−105^, respectively), supporting TGFβ/ENG signaling.

In addition, ENG may be affected by the BMP9-mediated pathway [12,13]. Pearson and Spearman correlation analyses between ENG and BMP9 in both blood and arteries were also performed. As results, we did not find a significantly positive correlation between ENG and BMP9 in the blood vessels (Supplementary Figure S1A,B, R = 0.03, *p*-value = 0.58 and R = −0.022, *p*-value = 0.69, respectively), implying there was no ENG/BMP9 regulation/connection in the blood vessels. Similar results showed no significantly positive correlations between ENG and BMP9 in the artery (Supplementary Figure S1C,D, R = 0.058, *p*-value = 0.15 and R = 0.075, *p*-value = 0.067, respectively), implying that no clear ENG-mediated regulation on BMP9 protein steady-state expression levels was evident, despite the well-known BMP9/ENG cooperation in the angiogenesis-signaling pathway.

## 4. Discussion

In three individuals affected by unrelated HHT1 pedigrees, McAllister et al. [4] first identified ENG gene mutations in 1994. In 1997, Pece et al. [40] described an ENG splice site mutation, resulting in the skipping of exon 3 and the absence of 47 amino acids in a newborn from an HHT1 family. Gallione et al. [38], in 1998, identified another 11 ENG mutations, including missense and splice site mutations in HHT1 families. Interestingly, in the French literature, Soukarieh et al. [41] recently identified two variations, c.-68G>A and c.-79C>T, at the 5′ untranslated region of ENG in unrelated HHT patients, shedding new light on the role of upstream open-reading frames in the expression regulation of ENG. In this study, an HHT1 Chinese pedigree was recruited, and whole-exome sequencing (WES) analysis, Sanger verification, and co-segregation were conducted. Western blotting was performed for the monitoring of ENG/VEGFα signaling. As a result, a nonsense, heterozygous variant for ENG/CD105: c.G1169A:p. Trp390Ter of the proband with HHT1 was identified, which was co-segregated with the disease in the M666 pedigree.

Moreover, all nonsense variants are expected to give rise to NMD, leading to the absence or significantly reduced levels of truncated ENG protein expression. That is one of reason why we did not observe the mutant form of the ENG variant (p.Trp390Ter) for the proband by Western blotting. Our RT-PCRs also failed to detect this variant of *ENG* in the blood.

Luzhou is located in Sichuan, Yunnan, Guizhou, and the Chongqing junction in southwest China, sites in the transition zone between the southern edge of the Sichuan Basin and the Yunnan–Guizhou Plateau, thus connecting the Yunnan–Guizhou Plateau in the south. This geographical location may have influenced the economic, cultural, and technological development of Luzhou, thus impacting the capacity of patients to seek effective diagnoses and treatments. In this study, an HHT pedigree with a 66-year-old female proband and eight family members was recruited from Luzhou in southwest China. The patient had shown repeated nasal and lip bleeding for more than 40 years, with this being aggravated for 10 years. She visited several local hospitals when she was young, and initially received diagnoses of hemorrhinia and/or hypertension. However, obtaining a definite clinical diagnosis from local hospitals proved challenging. It was only in July 2010 that the proband was first diagnosed as having hereditary telangiectasia at the outpatient department of the First Affiliated Hospital of the Third Military Medical University in Chongqing. Moreover, due to the great variabilities in phenotypes among HHT patients, gene diagnosis by WES was performed, and we successfully identified a heterozygous nonsense variant for ENG: c.G1169A:p. Trp390Ter in the proband. The parents of the proband may be unaffected as the proband claimed. Based on Curaçao’s criteria [16], this patient was primarily diagnosed with HHT, and was finally diagnosed as having HHT1 based on the results of gene diagnosis.

The ENG protein, together with ALK1 and the endothelial receptors of the TGFβ superfamily, is required for vascular integrity or angiogenesis [14]. Genetic studies on ENG in both mice and humans identified the critical role of the TGFβ pathway during angiogenesis. By binding to the type II receptor of TGFβ in endothelial cells, TGFβ activates two distinct type I receptors, ALK1 and ALK5, leading to opposing effects on the proliferation and migration of endothelial cells. Downregulated ENG expression disrupted the ALK1- and ALK5-dependent TGFβ pathways, disorganized the cytoskeleton, and led to a failure to form cord-like structures, thus leading to fragility in the small vessels and bleeding after injuries, which explains the clinical symptoms of the HHT disease. The VEGF level is also reported to correlate with TGFβ levels both in vivo and in vitro [42]. Then, we monitored the ENG and downstream gene VEGFα for angiogenesis via Western blotting, and the results revealed that the levels of the ENG protein levels in family members II:2, II:3, II:4, and III:2 were approximately doubled, while levels of the VEGFα protein showed approximately 3- to 4.5-fold increases, demonstrating that reduced ENG expression, due to the NMD-mediated degradation of the ENG variant (p.Trp390Ter)-carrying transcript, may result in reduced TGFβ/VEGFα expression in this Chinese family. Since similar results are expected for most ENG truncating mutations, as associated with NMD and a one-half reduction of ENG expression, future investigations are needed to confirm the connection between the ENG haploinsufficiency and VEGFα expression downregulation observed in the present study. We also noticed that the levels of the wild-type ENG protein in the proband were only half of those in their family members. Pearson and Spearman correlation analyses of the GTEx data of humans further suggested TGFβ/ENG/VEGFα signaling, implying ENG regulation in the blood vessels. Previous studies have shown that serum-measured circulating levels of VEGF seem to be increased in HHT patients [43,44]. The possible reasons underlying such a discrepancy were in agreement with the stimulation of VEGF synthesis proposed in the mouse model [43] or were unknown reasons. In addition, we cannot exclude the dysregulation of the BMP9-mediated pathways [12,13], rather than being strictly from TGFβ impairment, even though no correlation between ENG and BMP9 (*p* > 0.05) expression at the protein level was evident at GTEx expression. Of course, further study may be needed. Nevertheless, antithrombotic therapy (anticoagulation and antiplatelet therapy) is widely used for HHT patients but is poorly tolerated, with objectively higher morbidity or worsened bleeding. This often leads to premature dose-reduction or the discontinuation of the therapy in some patients or treatment regimens [45,46]. Therefore, ENG-pathway genes could be used as the therapeutic targets for HHT1 [47]. Interestingly, great progress has been made in developing drugs that target VEGF and the angiogenic pathway, for example, the anti-VEGF antibody bevacizumab [48,49,50].

Bleomycin, a glycopeptide and cytotoxic antibiotic, is a colorless or yellowish powder derived from natural products, isolated from *Streptomyces verticillus* (*S. verticillus* var. *Pingyangensis n.* sp. or *S. verticillus* var. *pingyangensis n.* var) by Chinese scholars in 1969 from a soil-fermentation broth in the Pingyang mountain area, Zhejiang Province of China. It is also called Pingyangmycin, and Pingyangmycin A2, A5, A6, B2, and B4 are the same as Bleomycin A2, A5, A6, B2, and B4, respectively [51]. This substance inhibits DNA metabolism, and is used as an antineoplastic agent, especially for treating solid tumors, including Hodgkin disease, lymphomas, testicular and germ-cell cancers, and tumors of the head and neck. Treatments with bleomycin, either alone or in combination with other agents, are often associated with mild to moderate serum enzyme elevations. Studies have used bleomycin to treat vascular malformations via sclerotherapy in both humans [52,53,54] and animals [55].

Interestingly, bleomycin has been reported to induce intracellular VEGFα mRNA and protein synthesis in pleural mesothelial cells (PMCs), and increase the secretory level of PMCs, both in animal models and in patients with idiopathic pulmonary fibrosis (IPF) [56]. Thus, bleomycin may be effective for use in HHT1 therapy by activating the above signaling pathways. In this regard, bleomycin (bleomycin a5 hydrochloride) was used on the proband (M666) in this study, combined with dexamethasone and lidocaine. After one week of treatment, the symptoms of the patient were relieved. There was no bleeding in the nasal cavity or lips within 6 months, and the quality of life of the patient was improved. This possible effective treatment may be based on compassion. However, this exceptional compassionate treatment needs be evaluated in a well-conducted therapeutic trial., the results of which would be of great interest to the medical community.

Sturge–Weber syndrome (SWS) is a rare, congenital, and sporadic neurovascular disease characterized by abnormal vasculature in the brain, eyes, and skin [57]. The variants of the *GNAQ* gene and the *GNA11* gene may be associated with SWS [58]. Sirolimus, a mammalian target of the rapamycin (mTOR) inhibitor, has been reported to be a potentially effective drug for the treatment of SWS [59,60,61]. Sirolimus may thus be a promising candidate for HHT1 treatment by targeting vascular anomalies. However, no report has so far shown sirolimus targeting TGFβ/VEGFα signaling.

## 5. Conclusions

In this study, we successfully identified a heterozygous variant for ENG: c.G1169A:p. Trp390Ter in a proband with HHT1 from a Chinese family. This nonsense disease-causing variant may result in deficient TGFβ/VEGFα signaling in the blood vessels, potentially rendering it beneficial for bleomycin treatments. Next-generation sequencing (NGS), including WES, should provide essential strategies for gene diagnosis, therapy, genetic counseling, and clinical management for rare genetic diseases, including that in HHT1 patients.

## Figures and Tables

**Table 1:** Biochemistry examination of the proband.

Contents	Value	Unit	Ranges
Total bilirubin (TBIL)	43.2↑	μmol/L	5.0–28.0
Direct bilirubin (DBIL)	15.7↑	μmol/L	<8.8
Indirect bilirubin (IBIL)	27.50↑	μmol/L	<20
Total bile acid (TBA)	5.5	μmol/L	<15

**Table 2:** Blood coagulation examination for the proband.

Contents	Value	Unit	Ranges
Prothrombin time (PT)	14.4	S	10~15
International normalized ratio (INR)	1.21		0.8~1.3
Prothrombin time ratio (PT-Ratio)	1.2		
Prothrombin time activity level (PT%)	76.5	%	70~130
Partial activated thromboplastin time (APTT)	27.2	S	22~40
Thrombin time (TT)	14.6	Sec	14~21
Fibrinogen (FIB)	4.14↑	g/L	2~4
D-dimer (D-Dimer)	250.22	µg/L	0~500
Fibrin degradation product (FDP)	1.31	mg/L	0~5

**Table 3:** Curaçao criteria (diagnostic criteria for HHT) for the proband.

Criterion	Description	Symptoms for the Proband (M66)
i. Epistaxis	Spontaneous and recurrent	The patient had recurrent spontaneous epistaxis starting at the age of about 26 years
ii. Telangiectasias	Multiple mucocutaneous telangiectasias at characteristic sites (e.g., fingers, tongue, lips, buccal mucosa)	The patient had mucosal telangiectasia in multiple parts of the tongue, lip, finger, and cheek
iii. Visceral lesions	Visceral AVMs ‡ in the lungs, liver, gastrointestinal tract, brain, and/or spine	The patient claimed that eating slightly hot food would lead to stomach bleeding, suggesting microvascular malformation, and abdominal color ultrasound indicated hepatic vascular malformation
iv. Family history	History of a first-degree relative who has been diagnosed with HHT according to the aforementioned criteria	No

Note—Unlikely diagnosis: fewer than two criteria are met. Probable diagnosis: two criteria are met. Definite diagnosis: three or four criteria are met. HHT: hereditary hemorrhagic telangiectasia; AVM: arteriovenous malformation.

## Data Availability

The data that support the findings of this study are available from the corresponding authors upon reasonable request. The data are not publicly available due to confidentiality.

## Supplementary Materials

The following supporting information can be downloaded at: https://www.mdpi.com/article/10.3390/genes15030304/s1. Figure S1: No effect of ENG variant on BMP 9 expression. A&B Pearson and Spearman correlation analysis of the genes between ENG and BMP 9 in blood of GTEx expression data, respectively. C&D Pearson and Spearman correlation analysis of the genes between ENG and BMP 9 in arteries (aorta, coronary, and tibial) of GTEx expression data, respectively. To perform Pearson and Spearman analyses via GEPIA (http://gepia2.cancer-pku.cn/#index)correlation (accessed on 21 January 2024) on GTEx expression data, we computed the genetic correlations between ENG and BMP 9 in blood and the artery (aorta, coronary, and tibial) respectively *p* < 0 05 was considered to be significant.
