# Supplementary material for: The ENG/VEGFα Pathway Is Likely Affected by a Nonsense Variant of Endoglin (ENG)/CD105, Causing Hereditary Hemorrhagic Telangiectasia Type 1 (HHT1) in a Chinese Family
Source: Genes (Basel). 2024 Feb 27;15(3):304. doi: 10.3390/genes15030304 (PMC10970080; doi:10.3390/genes15030304)
Supplement: Supplementary file 1 [file genes-15-00304-s001.zip › genes-2880594-supplementary.pdf]

Supplementary figures

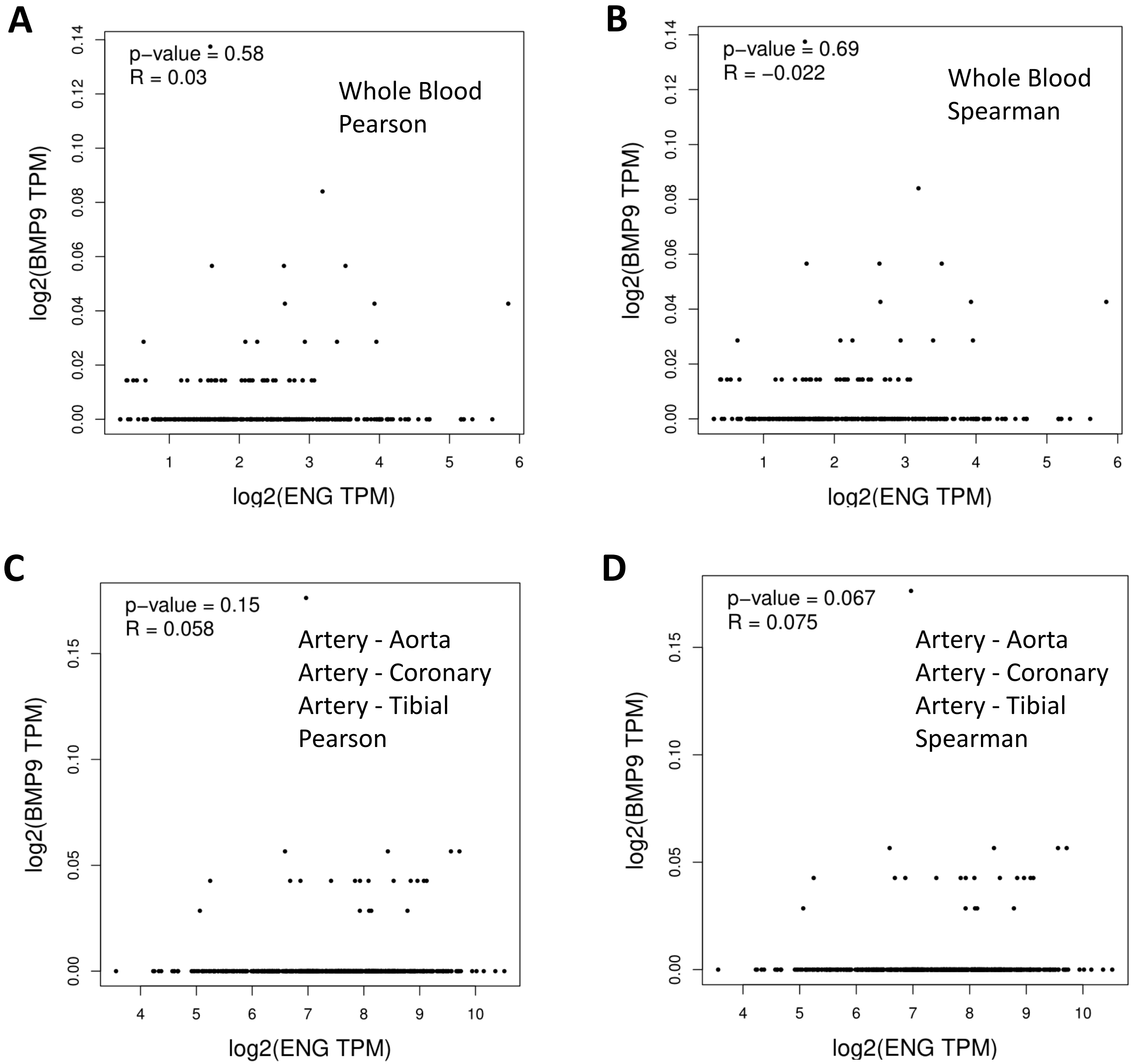

Supplementary Figure S1. No effect of ENG variant on BMP9 expression. A&B. Pearson and Spearman correlation analysis of the genes between ENG and BMP9 in blood of GTEx expression data, respectively. C&D. Pearson and Spearman correlation analysis of the genes between ENG and BMP9 in arteries (aorta, coronary, and tibial) of GTEx expression data, respectively.

To perform Pearson and Spearman analyses via GEPIA (http://gepia2.cancer-pku.cn/#correlation) on GTEx expression data, we computed the genetic correlations between ENG and BMP9 in blood and the artery (aorta, coronary, and tibial), respectively. $P<0.05$ was considered to be significant.
